# Cutaneous Manifestations of Malaria and Their Prognostic Windows: A Narrative Review

**DOI:** 10.7759/cureus.41706

**Published:** 2023-07-11

**Authors:** Christopher S Farkouh, Parsa Abdi, Faiza Amatul-Hadi, Michelle R Anthony, Qaisar Ali Khan, Kyla Manja, Christian Manja, Syed Masood Ali

**Affiliations:** 1 Dermatology, Rush Medical College, Chicago, USA; 2 Faculty of Medicine, Memorial University of Newfoundland, St. John's, CAN; 3 Dermatology, Mercer University School of Medicine, Macon, USA; 4 Pathology, University of Arizona College of Medicine, Tucson, USA; 5 Internal Medicine, Khyber Teaching Hospital, Peshawar, PAK; 6 Internal Medicine, District Headquarter Teaching Hospital, Kohat, PAK; 7 General Medicine, University of Pennsylvania, Philadelphia, USA; 8 Family Medicine, University of Illinois College of Medicine at Peoria, Peoria, USA

**Keywords:** dermatology, plasmodium falciparum, narrative review, cutaneous manifestations, malaria

## Abstract

Malaria is a vector-borne tropical infection caused by protozoa of the genus *Plasmodium* and is transmitted by the bite of an infected Anopheles mosquito. The disease is commonly characterized by fever, edema, thrombocytopenia, hypoglycemia, anemia, and myalgias; however, the infection’s cutaneous presentations are not commonly emphasized and tend to be overlooked.

A literature search was conducted that focused on the various skin pathologies that malaria patients have been noted to present with using case reports and currently available literature. We describe the various skin manifestations associated with malaria, such as purpura fulminans, febrile urticaria, cutaneous leishmaniasis co-infections, urticaria infectiosum, vivax-induced severe thrombocytopenia petechiae, acral skin necrosis, and reticulated erythema, and how each of these skin manifestations may provide insight into the patient’s prognosis.

Documentation and vigilance regarding these cutaneous manifestations must be emphasized as they may lead to better patient outcomes and a stronger understanding of the patient’s underlying malaria.

## Introduction and background

Malaria, a blood-borne tropical infection caused by protozoa of the genus *Plasmodium*, is transmitted by the bite of an infected Anopheles mosquito. Following invasion and growth within erythrocytes once inside the human body, the *Plasmodium* parasite causes the disease's periodic fever episodes and systemic symptoms. Malaria is a significant global concern since it affects 10,000 to 30,000 of the 125 million visitors to endemic regions yearly, with a 1% mortality rate [1]. An initial evaluation of unknown general fever in stable individuals with suspected malaria exposure entails a comprehensive metabolic panel, coagulation tests, blood cultures, a complete blood count, and chest imaging, given that the disease is characterized by fever, edema, myalgias, thrombocytopenia, hypoglycemia, and anemia [2]. Malaria has been associated with various complications including liver or renal impairment, cerebral malaria, acute respiratory distress syndrome, and spontaneous splenic rupture [3]. Malaria’s cutaneous presentations, however, can be commonly overlooked in favor of focusing on the more conventional, systemic manifestations of infection. Malaria can present with cutaneous lesions, such as angioedema, petechiae, gangrene, and urticaria [1]; however, rarer manifestations have been reported in the literature. Healthcare workers in endemic locations must be familiar with the many cutaneous malaria symptoms as these skin conditions can reveal important information about the severity of an illness, the effectiveness of treatment, and future repercussions. Clinicians in non-endemic locations should also be familiar with the cutaneous aspects of malaria to aid in early detection and appropriate care in light of the globalization of travel and growing migration trends.

Objectives

Our narrative review details malaria’s relationship with its various skin pathologies, some less common than others, and strives to illuminate the prognostic value of the infection’s cutaneous manifestations.

Methods

A literature search was performed using PubMed, MEDLINE, Scopus, and Google Scholar. Through the use of case reports, narrative reviews, and systematic reviews, our investigation focused on the various skin pathologies that patients afflicted with malaria have presented with. Articles were selected and narratively reviewed by two reviewers. A third reviewer resolved any incongruity in the initial article selection process. A total of 11 cases were ultimately selected to be included in this analysis.

Seven malarial manifestations were assessed using a severity index (SI) score based on four metrics, namely “cutaneous findings,” “functional outcomes,” “degree of sepsis,” and “time to resolution.” The SI was calculated using an evaluation matrix, where each metric received a weighting out of three based on the severity associated with malaria. Specifications of weighting for each metric are given in Table 1.

**Table 1:** Evaluation matrix containing scale weights attributed to the categories of cutaneous findings, functional outcomes, degree of sepsis, and time to resolution BP, blood pressure

Weighting	Cutaneous findings	Functional outcomes	Degree of sepsis	Time to resolution
1	Non-necrotic	Full recovery	Stable (fever only)	Order of months
2	Thrombocytopenic bleeding	Significant disability	Hemodynamically stable but possessed some level of organ/systems dysfunction	Order of weeks
3	Necrotic	Bedridden	Hypotensive shock (low BP, cold extremities)	Order of days

## Review

Purpura fulminans

Purpura fulminans (PF) is a condition characterized by disseminated intravascular coagulation (DIC) that commonly occurs in patients with an acquired deficiency in anticoagulants, often due to a septic infection [3]. Historically, its development is suggestive of a poor prognosis as impairment of the protein C anticoagulation pathway is implied. Commonly suspected organisms include gram-negative cocci, gram-positive bacteria, and parasites such as malaria [4]. *Plasmodium falciparum* infection is noted to be associated with low levels of protein C, protein S, tissue plasminogen activator, and antithrombin with an increased level of plasminogen activator inhibitor-1 [4,5].

Chaudhary et al. reported a case of a 22-year-old man with rigor, fever, and chills for seven days who presented with bluish-black discoloration of both feet and legs to a hospital in South East Asia [6]. The patient’s hemoglobin was 9.3 g/dL, leukocyte count was 12,800/mm^3^ (with a breakdown of 77% polymorphs, 20% lymphocytes, 1% eosinophils, and 2% monocytes), and had a decreased platelet count (19,000/mm^3^). Additionally, he had moderately elevated liver enzymes, an increased prothrombin time (25.9 seconds), and a blood smear positive for *P. falciparum* antigen [6]. The patient was diagnosed with complicated malaria. He was placed on intravenous artesunate, low molecular weight heparin, and warfarin as treatment. The patient’s fever gradually resolved by day 3, as did his pedal edema, PF, and dark discoloration of his lower limbs.

Another report describes a case of a 51-year-old Caucasian male with a past medical history of post-traumatic splenectomy who had just returned from a vacation in Madagascar [7]. He had no antimalarial chemoprophylaxis and presented with fever and asthenia upon admission. His symptoms and vitals included hypotension (60/40 mmHg), a temperature of 101.6°F, cold extremities, and extensive necrotic and purpuric skin lesions on his hands and legs. Further hematological analysis revealed a peripheral blood smear indicative of *P. falciparum*. He received intravenous fluid, norepinephrine, low-dose hydrocortisone, intravenous quinine, vitamin K supplementation, and ceftriaxone, and recovered gradually. However, the purpura progressed to skin necrosis of the feet, and both were amputated two months later.

PF is characterized by symmetrical distal ischemic damage and hemorrhagic infarction of two or more locations without obstructing large vessels [8]. The aforementioned hemorrhagic skin lesion presentation coupled with DIC, travel history to an endemic malaria area, and typical blood smear point to the diagnosis of PF due to *P. falciparum *malaria. PF can be fatal, often requiring eventual amputations; thus, early recognition is paramount.

PF received an SI-weighted score of 9 for “cutaneous finding,” 6 for “degree of sepsis,” 9 for “time to resolution,” and 3 for “functional outcome,” giving the cutaneous manifestation a total SI score of 27.

Febrile urticaria

A classic presentation of febrile urticaria in the pediatric population is the appearance of wheals associated with edema of underlying subcutaneous tissues. This skin manifestation is believed to result from the malarial subtypes *P. falciparum* and *Plasmodium vivax* [9,10]. It is thought that this skin manifestation is antigen-implicated - involving IgE mediation and/or mast cell degranulation processes [9]. Scientists speculate that the process of Plasmodial daughter merozoites attaching to specific erythrocyte receptors yields an antigenic response by altering the membranes of red blood cells. The resulting increase in the antigenicity of the red blood cells may lead to the degranulation of large stores of mast cells, causing red, itchy wheals [9]. In these patients, the resulting inflammatory markers released lead to the classic presentation of bronchospasm, shock, and angioedema [10].

Zaki and Shanbag described a case involving a nine-year-old Indian girl with a high-grade fever and bilateral upper and lower limb pruritic urticarial rashes [11]. Peripheral blood smear displayed trophozoites of *P. vivax*. She was successfully treated within three days without complications with chloroquine and antihistamines. The patient additionally received a 14-day course of primaquine for radical cure. A second case study by Sharma et al. described a family where three members, on separate occasions, presented with urticarial lesions that occurred concomitantly with a fever secondary to *P. falciparum* infection, suggestive of a potential genetic predisposition nature of febrile urticaria [10]. It was found that one of the family members, a 15-year-old male, was successfully treated with intravenous artesunate and oral doxycycline, which improved his skin conditions and eventually resulted in a full recovery over two days.

Febrile urticaria received an SI-weighted score of 3 for “cutaneous finding,” 2 for “degree of sepsis,” 3 for “time to resolution,” and 1 for “functional outcome,” giving the cutaneous manifestation a total SI score of 9.

Malaria and cutaneous leishmaniasis coinfection

Malaria and cutaneous leishmaniasis (CL) are two of the world’s most prominent vector-borne parasitic diseases affecting developing nations [12,13]. Malaria and CL are jointly endemic in tropical countries in large regions, which may influence the evolution of host-parasite interactions. Interestingly, malaria-CL joint infection outcomes differ depending on the specific subspecies of leishmania involved in the infection [13,14]. It is speculated that alterations in cytokine levels and phenotypic dynamics of thymic/splenic T cells are closely related to the immune response when malaria and CL are jointly implicated [14]. Changes in serum cytokine levels and thymus dynamics during infection appear to be closely related to these alterations in malaria and CL progression [15].

One case report details a rare case of a child, age 5, who exhibited black necrotic lesions, skin darkening, and fever in Nepal [15]. The young boy presented with a history of on-and-off high-grade fever associated with chills and rigor, abdominal pain, and constipation for three months. In addition to his febrile state and hepatosplenomegaly, the patient also displayed pancytopenia [15]. His hemoglobin was 7.2 g/dL, red blood cell (RBC) count was 2.94 x 10^9^/L, white blood cell count was 2.90 x 10^9^/L, and platelet count was 63 x 10^9^/L. An intracellular Leishmania-Donovan body and schizonts of *P. vivax* were observed on peripheral blood smear, as well as a positive RK 39 immunochromatographic and malaria rapid diagnostic test, further corroborating the diagnoses [15]. A total of three doses of chloroquine phosphate were used in conjunction with an infusion of free liposomal amphotericin B (10 mg/kg), leading to a full recovery over 15 days. After a follow-up blood film, the patient's clinical condition had significantly improved, and the child was clear of their prior parasitic infection [15].

A cross-sectional study conducted in Ethiopia assessed the prevalence of coinfection of visceral leishmaniasis (VL) with malaria [16]. A total of 384 VL patients were recruited, with 83 cases of VL reported (21.6%) and 45 cases of malaria reported (11.7%). It was found that 40 (89%) of malaria cases were positive for *P. falciparum*, 5 (11%) were positive for *P. vivax*, and a total of 16 (4.2%) individuals were found to be coinfected with VL and malaria. In the interest of further exploring potential etiologies of this joint infection, it was estimated that 188 (46.9%) study participants traveled in the past, with 10 (5.6%) of these individuals presenting with a combined VL-malaria infection.

CL coinfection received an SI-weighted score of 3 for “cutaneous finding,” 4 for “degree of sepsis,” 6 for “time to resolution,” and 1 for “functional outcome,” giving the cutaneous manifestation a total SI score of 14.

Plasmodium vivax malaria presenting as urticaria infectiosum

Although rare, cutaneous malaria involvement can manifest as severe urticaria and angioedema [17]. The most characteristic lesions of this type of urticaria include multiple, large itchy wheals and angioedema caused by activation and degranulation of mast cells in the skin with subsequent release of vasodilatory mediators that lead to downstream sensory nerve stimulation [18]. When merozoites are discharged into circulation, they adhere to particular erythrocyte receptors, which alter the red cell membrane by revealing the previously concealed surface antigens. The extensive mast cell degranulation may have been a result of the RBCs' enhanced antigenicity, which was either brought on by plasmodial infection or independent plasmodial antigen stimulation [18].

Kapse reports a case of vivax malaria that presented clinically as urticaria and angioedema [19]. A seven-year-old male child presented with swelling, fever, and numerous itchy wheals distributed across the entire body. Further examination revealed that the patient also showed perioral and periorbital angioedema. He was subsequently diagnosed with acute urticaria. However, despite receiving hydroxyzine and antipyretic medication, his symptoms persisted, and urticaria spread. A second peripheral smear taken during a subsequent feverish episode revealed *P. vivax* malaria trophozoites, contrary to the previous negative initial peripheral smear. After starting the patient on oral chloroquine, his health dramatically improved within three days, resulting in his fever and the urticarial rash resolving.

Furthermore, 10 individuals who presented with urticaria as a manifestation of malaria, either with or without fever, were described by Godse and Zawar [20]. All the patients in the study had malarial trophozoites in their blood and were undergoing treatment with antimalarial medications, which ultimately diminished their urticaria. The urticarial resistance to antihistamines and the infection’s improvement after receiving antimalarial medication support the presumed diagnosis of malaria-induced urticaria. The presence of widespread urticaria and fever should alert medical professionals to a potential underlying malarial infection and prompt necessary examinations to reduce malaria-related morbidity and mortality.

Urticaria infectiosum received an SI-weighted score of 3 for “cutaneous finding,” 2 for “degree of sepsis,” 3 for “time to resolution,” and 1 for “functional outcome,” giving the cutaneous manifestation a total SI score of 9.

Plasmodium vivax malaria with severe thrombocytopenia petechiae and varied cutaneous manifestations

Malaria is known to cause mild thrombocytopenia during its course of infection. In some cases, however, the extent of platelet drop can be rather severe. In one such instance, a four-year-old girl from India experienced severe thrombocytopenia, which manifested as cutaneous and mucosal bleeding [21]. The case involved six days of fever, chills, and other systemic signs, with 36 hours of hematuria before the presentation. The patient had no prior history or family history of bleeding disorders. On admission, the girl had significant petechial bleeding on her lower extremities. Her platelet count was approximately 11,000/mm^3^, and she was hypotensive with signs of hypovolemic shock. Her hemoglobin was low at 7.2 g/dL, she displayed mild hepatosplenomegaly, and her antigen and PCR testing were positive for *P. vivax* infection. Coagulation assays were in the normal range, including prothrombin time, activated partial thromboplastin time, fibrinogen count, and D-dimer levels. Platelet transfusion was attempted but failed to raise the patient's platelet count. Subsequent malarial treatment with parenteral artesunate, oral mefloquine, and primaquine was initiated, leading to fever resolution and rapid clearance of the malarial parasite. Intravenous immunoglobulin (IVIG) was begun in an attempt to address thrombocytopenia and bleeding. Platelets 10 days post-IVIG infusion was 74,000 and returned to an average count at six weeks. Given the rapid and successful response to IVIG, it is speculated that epitope mimicry between the *P. vivax* parasite and platelets might have been the driving mechanism behind the patient’s thrombocytopenia and bleeding [22].

*Plasmodium* *vivax* malaria with severe thrombocytopenia petechiae received an SI-weighted score of 6 for “cutaneous finding,” 6 for “degree of sepsis,” 6 for “time to resolution,” and 1 for “functional outcome,” giving the cutaneous manifestation a total SI score of 19.

Acral skin necrosis in patients with malaria

Superficial skin necrosis of the extremities may arise from local trauma or microvascular obstruction. This condition is abnormal in younger patients and has been associated with atrial fibrillation, myocardial infarction, aneurysm, endocarditis, cryoglobulinemia, and vasculitis [23]. Dos Santos et al. reported a case of acral skin necrosis in a 21-year-old male associated with *P. falciparum* malaria [24]. The patient had anemia, low platelets, jaundice, disorientation, high parasitemia, hypoglycemia, and black necrotic maculae that were encircled by an erythematous halo seen exclusively on the toes. Following treatment for malaria, the clinical course went well, and the skin lesions improved without any long-term effects over seven days. The severity of malaria, in this case, was suggested by the presence of altered consciousness, hypoglycemia, hemolytic jaundice, thrombocytopenia, and elevated serum lactate dehydrogenase.

The association of acral necrosis with *Plasmodium *malarial infection is not well documented. In the case presented by Dos Santos et al., the etiology of these lesions may be hypothesized to be due to the increased adherence of *Plasmodium*-parasitized erythrocytes to endothelial cells in the microvascularization of the lower limbs [24].

Acral skin necrosis received an SI-weighted score of 6 for “cutaneous finding,” 4 for “degree of sepsis,” 3 for “time to resolution,” and 1 for “functional outcome,” giving the cutaneous manifestation a total SI score of 14.

Reticulated erythema in malaria

Reticulated erythema is a clinical description that describes a skin rash with a "net-like," "chicken wire," or "sieve-like" pattern. The rash is associated with systemic, infectious, acquired, and congenital dermatoses [25]. Two case reports from India and one from Morocco report the presence of reticulated erythema in malaria in three patients [26,27]. The three reported cases of reticulated erythema, interestingly, all occurred in children and adolescents. Vaishnani described the first case of reticulated erythema with malaria in a 19-year-old Indian male [26], who presented with typical signs of malaria, including fever, tachycardia, hypotension, and headache. Skin examination was significant for blotchy reticulated erythema on the upper and lower extremities. Peripheral smear confirmed the presence of *P. falciparum* trophozoites. The patient was treated with antimalarial drugs α-β arteether, quinine, supportive platelet concentrate, and intravenous fluids. The patient's rash responded well on day 3 of treatment and was resolved by day 9 [26]. Vaishnani also described the case of a six-year-old Indian male with fever, weakness, and hepatosplenomegaly [26]. The patient had non-itchy reticulate erythema involving the upper extremities with associated petechiae. After confirmation of *P. falciparum* malaria, physicians treated him with α-β arteether, supportive platelet concentrates, and intravenous fluids. His rash improved by day 7.

The aforementioned case report from Morocco involved a 20-year-old female admitted with an acute febrile rash [27]. Her physical examination was positive for reticulated erythema involving the trunk and urticaria of the upper extremities. Additionally, the patient displayed a positive peripheral smear for *P. falciparum*, resulting in her treatment with chloroquine and antihistamine. The patient's rash responded appropriately to the medications and resolved without further complications. It is proposed that erythema and urticaria occur due to the release of histamine, leukotrienes, and platelet-activating factor. Notably, the associated petechiae seen in the first two causes are suspected to be due to an immune complex resulting in vessel damage [27].

Reticulated erythema received an SI-weighted score of 3 for “cutaneous finding,” 4 for “degree of sepsis,” 3 for “time to resolution,” and 1 for “functional outcome,” giving the cutaneous manifestation a total SI score of 19.

The comprehensive summary of the cutaneous manifestations, malaria types, interventions, outcomes, and major clinical findings of each of the cases presented in this review are given in Table 2.

**Table 2:** The populations, interventions, outcomes, and major clinical findings of each malaria-associated cutaneous manifestation.

Cutaneous manifestation	Cases	Malaria type	Intervention	Outcome (time to resolution)	Major clinical findings
Purpura fulminans	Patient 1 [6]	*Plasmodium falciparum*	Intravenous artesunate, low molecular weight heparin, and warfarin	Resolution (3 days)	Bluish-black discoloration of bilateral lower extremities
	Patient 2 [7]	*Plasmodium falciparum*	Intravenous fluid, norepinephrine, low-dose hydrocortisone, intravenous quinine, vitamin K supplementation, and ceftriaxone	Progression to skin necrosis and amputation	Extensive necrotic and purpuric skin lesions of hands and bilateral lower extremities
Febrile urticaria	Patient 1 [11]	*Plasmodium vivax*	Chloroquine and antihistamines followed by a 14-day course of primaquine	Resolution (3 days)	Bilateral upper and lower limb itchy urticarial rashes
	Patient 2 [10]	*Plasmodium falciparum*	Intravenous artesunate and oral doxycycline	Resolution (2 days)	Urticarial lesions in unspecified locations
Cutaneous leishmaniasis coinfection	Patient 1 [15]	*Plasmodium vivax*	Chloroquine phosphate, liposomal amphotericin B, and primaquine phosphate	Resolution (15 days)	Kala-azar, necrotic lesions
Urticaria infectiosum	Patient 1 [19]	*Plasmodium vivax*	Chloroquine	Resolution (3 days)	Diffuse, itchy wheals
Thrombocytopenia petechiae	Patient 1 [21]	*Plasmodium vivax*	Parenteral artesunate, oral mefloquine, primaquine, and intravenous immunoglobulin	Resolution (6 days)	Petechial bleeding of lower extremities
Acral skin necrosis	Patient 1 [24]	*Plasmodium falciparum*	Unspecified malaria treatment	Resolution (7 days)	Black necrotic maculae encircled by an erythematous halo on toes
Reticulated erythema	Patient 1 [26]	*Plasmodium falciparum*	α-β arteether, quinine, supportive platelet concentrate, and intravenous fluids	Resolution (9 days)	Blotchy reticulated erythema on the upper and lower extremities
	Patient 2 [26]	*Plasmodium falciparum*	α-β arteether, supportive platelet concentrates, and intravenous fluids	Resolution (7 days)	Non-itchy reticulated erythema involving the upper extremities with associated petechiae
	Patient 3 [27]	*Plasmodium falciparum*	Chloroquine and antihistamine	Resolution (not disclosed)	Reticulated erythema involving the trunk and urticaria of the upper extremities

Malarial severity analysis

The malarial severity analysis revealed SI scores ranging from 9 to 27, with a maximum score of 27 obtainable from the matrix. The SI scores for the cutaneous manifestations of malaria (including PF), thrombocytopenia petechiae, acral skin necrosis, CL coinfection, reticulated erythema, urticaria infectiosum, and febrile urticaria scored 27, 19, 14, 14, 11, 9, and 9, respectively, as shown in Table 3.

**Table 3:** Final weights ascribed to each malaria-associated cutaneous manifestation based on SI SI, severity index; WS, weighted score

Criteria	Weight	Purpura fulminans		Febrile Urticaria		Cutaneous leishmaniasis coinfection		Urticaria infectiosum		Thrombocytopenia petechiae		Acral skin necrosis		Reticulated erythema	
		Rating	WS	Rating	WS	Rating	WS	Rating	WS	Rating	WS	Rating	WS	Rating	WS
Cutaneous findings	3	3	9	1	3	1	3	1	3	2	6	2	6	1	3
Degree of sepsis	2	3	6	1	2	2	4	1	2	3	6	2	4	2	4
Time to resolution	3	3	9	1	3	2	6	1	3	2	6	1	3	1	3
Functional outcomes	1	3	3	1	1	1	1	1	1	1	1	1	1	1	1
Total SI		27		9		14		9		19		14		11	

## Conclusions

Malaria's cutaneous manifestations indicate the degree of malaria infection and provide insight into an individual's prognosis. By using a total SI to assess cutaneous findings, functional outcomes, degree of sepsis, and time to resolution, we can better analyze a patient’s cutaneous presentation of malaria. PF yielded the highest total SI weight at 27, followed by P. vivax malaria with severe thrombocytopenia petechiae (19), acral skin necrosis (14), CL coinfection (14), reticulated erythema (11), febrile urticaria (9), and urticaria infectiosum (9). Given the rare nature of these various malaria-associated skin pathologies, it can be quite difficult to truly ascertain accurate presentations of these cutaneous presentations as they vary from individual to individual. Had there been more literature on similar incidences of these cases, a more accurate depiction of the affliction could have been provided, leading to more accurate insights into the prognoses of the aforementioned manifestations. It is of the utmost importance that physicians stay vigilant of these possible, rare, cutaneous presentations as focusing solely on objective lab values may prevent optimal and early treatment measures.
